# Investigation on Single-Molecule Junctions Based on Current–Voltage Characteristics

**DOI:** 10.3390/mi9020067

**Published:** 2018-02-02

**Authors:** Yuji Isshiki, Yuya Matsuzawa, Shintaro Fujii, Manabu Kiguchi

**Affiliations:** Department of Chemistry, Graduate School of Science, Tokyo Institute of Technology, 2-12-1 W4-10 Ookayama, Meguro-ku, Tokyo 152-8551, Japan; isshiki.y.ab@m.titech.ac.jp (Y.I.); matsuzawa.y.ad@m.titech.ac.jp (Y.M.)

**Keywords:** single-molecule junction, current–voltage characteristics, atomic and electronic structure, vibrational mode, spin state

## Abstract

The relationship between the current through an electronic device and the voltage across its terminals is a current–voltage characteristic (*I*–*V*) that determine basic device performance. Currently, *I*–*V* measurement on a single-molecule scale can be performed using break junction technique, where a single molecule junction can be prepared by trapping a single molecule into a nanogap between metal electrodes. The single-molecule *I*–*V*s provide not only the device performance, but also reflect information on energy dispersion of the electronic state and the electron-molecular vibration coupling in the junction. This mini review focuses on recent representative studies on *I*–*V*s of the single molecule junctions that cover investigation on the single-molecule diode property, the molecular vibration, and the electronic structure as a form of transmission probability, and electronic density of states, including the spin state of the single-molecule junctions. In addition, thermoelectronic measurements based on *I*–*V*s and identification of the charged carriers (i.e., electrons or holes) are presented. The analysis in the single-molecule *I*–*V*s provides fundamental and essential information for a better understanding of the single-molecule science, and puts the single molecule junction to more practical use in molecular devices.

## 1. Introduction

Using single molecules as active components in electronic devices is one of the human dreams. To realize the single-molecule device, a single-molecule junction, which is the basic unit of molecular device, has been extensively studied since the first discovery in 1974 [1,2,3,4,5]. The single-molecule junction can be fabricated by using the break junction technique [6,7]. In the presence of target molecules, a metal atomic contact is mechanically broken. After breaking the metal atomic contact, a nano gap is formed between metal electrodes. If molecules diffuse to the nanogap and bridge the metal electrodes, a single-molecule junction is formed. Currently, the electronic conductance of the single-molecule junctions has been investigated for various molecules from simple molecule (e.g., hydrogen, oxygen) [8,9] to complex molecules (e.g., supramolecule, polymer) [10,11,12]. Memory, diode, switch, and other functional properties are reported for the single-molecule junctions [13,14,15,16,17,18]. The single-molecule measurement techniques have been applied to the sensors for biomolecules, such as DNA, RNA, and protein [19,20,21,22,23,24].

The atomic and electronic structures and spin state of the single-molecule junction is the essential information to understand the properties of the single-molecule junction. The investigation on electronic current passing through the single molecule bridging metal electrodes is a direct and efficient strategy to study the single-molecule junction. The detailed evaluation on the current–voltage characteristic provides the information (e.g., diode characteristics, atomic and electronic structure, spin state) about the single-molecule junction [8,25,26]. In this mini review, we discuss the analysis of diode characteristics, atomic and electronic structure, and spin state of the single-molecule junction using the current–voltage characteristics.

## 2. Diode Characteristics

**Figure 1:**
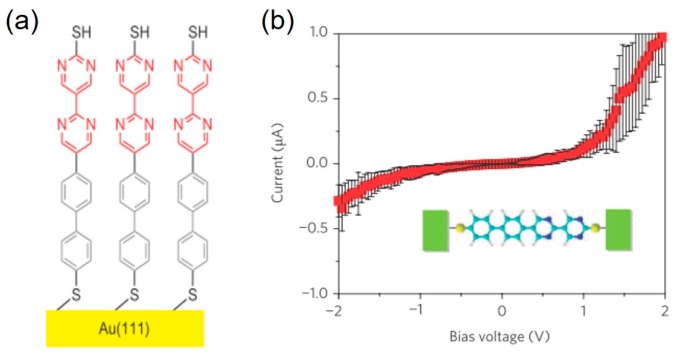
(**a**) The molecular diode with the diblock molecule. An electron deficient bipyrimidinyl unit is bound to an electron rich biphenyl unit via a covalent bond. (**b**) Average *I*–*V* curves of the single-molecule junctions of the diblock molecule. Reproduced with permission from [15], copyright Nature Chemistry Group 2009.

The diode characteristic of the single-molecule junction can be studied by examining the symmetry of the *I*–*V* curve. Tao’s group succeeded in creating the single molecular diode using the diblock molecule, in which an electron deficient bipyrimidinyl (acceptor) unit is bound to an electron rich biphenyl (donor) unit via a covalent bond [15]. The key technique for the single-molecule diode is the control of the molecular orientation. They terminated the diblock molecule with two different protecting groups of trimethylsilylethyl (for dipyrimidinyl unit) and cyanoethyl (for diphenyl unit). First, the cyanoethyl protecting group was de-protected, which exposed the thiol group at the diphenyl unit. The diphenyl end was bound to the Au substrate via this unprotected thiol group. Second, the trimethylsilylethyl protecting group was removed, which exposed the thiol group at the dipyrimidinyl unit. The approaching tip to the molecule caused the bridging of the single molecule between tip and substrate with a fixed orientation: tip–dipyrimidinyl–biphenyl–substrate junction. Figure 1 shows the *I*–*V* curve of single diblock molecular junction. Electrons preferentially flow from bipyrimidinyl unit (acceptor) to biphenyl unit (donor). The diode characteristics are clearly observed for the single-molecule junction. The average rectification ratio is about five at the bias voltage of 1.5 V.

**Figure 2:**
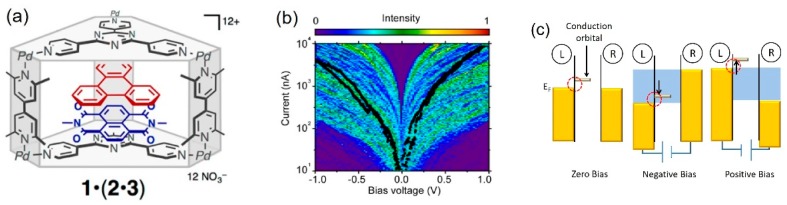
The single-molecule diode with the heterocomplex. (**a**) The naphthalenediimide and triphenylene are accommodated in the columnar cage; (**b**) Distribution of *I*–*V* curves of the heterocomplex single-molecule junctions, constructed from 500 *I*–*V* traces. Black lines are examples of *I*–*V* curves; (**c**) Energy diagram of the single-molecule diode. The conduction orbital is lowest unoccupied molecular orbital (LUMO), and the electronic coupling between LUMO and left electrode is larger than that of the right electrode. Reproduced with permission from [27], copyright Journal of the American Chemical Society Group 2015.

The diode characteristics have been observed for host–guest systems, where the guest molecules are included in a molecular cage molecule [27]. The number and orientation of the accommodated guest molecules can be controlled by using the cage. In addition, the guest molecules are noncovalently embedded in the cage, and thus, they can be replaced by other aromatic molecules. Fujii et al. used the heterocomplex, where naphthalenediimide (acceptor) and triphenylene (donor) were included in the cage, as the molecular diode. Figure 2 shows distribution of the *I*–*V* curves of the heterocomplex single-molecule junctions. The black curves are examples of the *I*–*V* curves, exhibiting an asymmetric shape. The larger current flows at the positive bias voltage. The average rectification ratio is about five at the bias voltage of 0.5 V. Theoretical analysis reveals that this diode characteristic is caused by the stacking order of the encapsulated aromatic molecules against the direction of electron transport*.* In the case of the heterocomplex single-molecule junction, the conduction orbital is lowest unoccupied molecular orbital (LUMO). The LUMO is localized on the naphthalenediimide unit*,* and it effectively hybridizes with the metal electrode (left electrode in Figure 2c). Due to the asymmetric strength of the metal–molecule coupling at the interface, the conduction orbital (LUMO) follows the movement of the Fermi level of the left metal electrode. When the positive bias is applied to the left electrode, the LUMO is shifted within the bias window. Electron transport through this molecular orbital leads to large currents. Meanwhile, when the negative bias voltage is applied to the left electrode, the LUMO is pushed away from the bias window, which causes the current suppression. Currently, a variety of single-molecule diodes have been reported, and the rectification ratio of 200 is reported for the single-molecule junction with the oligomer of thiophene-1,1-dioxide in ionic liquid [28,29,30].

## 3. Vibrational Mode

The interaction between molecular vibration and conduction electrons can cause a change in the conductance of the single-molecule junction [2]. At the low bias voltage, electrons elastically tunnel through the junction. Above a threshold voltage, where *hν* = *eV*, molecular vibrations can be excited by conduction electrons, and electrons are inelastically scattered. The threshold voltage corresponds to the vibrational energy, and thus, the vibrational information can be obtained by measuring the differential conductance (*dI*/*dV*) as a function of bias voltage. Since the conductance change induced by the excitation of vibrational mode is small (several %), the differential conductance is measured with the lock in amplifier, in order to detect the weak signal.

**Figure 3:**
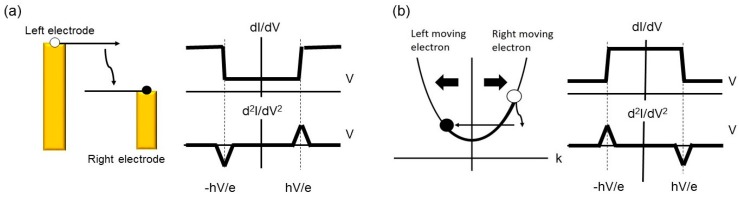
(**a**) Schematic image of inelastic electron tunneling spectroscopy (IETS). The excitation of a vibrational mode by conduction electrons opens an additional inelastic channel together with the elastic channel. Above a threshold voltage (*V* = *hν*/*e*), the conductance increases, and peaks are observed in *d*^2^*I*/*dV*^2^ curve; (**b**) Point contact spectroscopy (PCS): The electrons moving the right direction are scattered backwards by the vibrational mode excitation. Above the threshold voltage, the conductance decreases, and dips are observed in *d*^2^*I*/*dV*^2^ curve.

The direction of the conductance change induced by excitation of vibrational mode depends on the conductance of the junction [31]. At the low conductance regime (tunneling regime), the differential conductance is enhanced at the threshold voltage, while the differential conductance is suppressed at the high conductance regime (contact regime). The differential conductance measurement at the tunneling regime is named inelastic electron tunneling spectroscopy (IETS), and the differential conductance measurement at the contact regime is named point contact spectroscopy (PCS). The mechanisms of IETS and PCS are shown in Figure 3. In the tunneling regime, excitation of vibrational mode corresponds to the opening of the additional inelastic channel, together with the original elastic channel. The differential conductance of the single-molecule junction increases by the opening of this inelastic channel. The conductance enhancement is noted in the derivative of the differential conductance, *d*^2^*I*/*dV*^2^. In *d*^2^*I*/*dV^2^* curve, a peak and a dip are detected at the positive and negative bias voltage, respectively. In the contact regime, electrons are delocalized over the single-molecule junction. The transport of electrons can be examined in the momentum space. When the positive voltage is applied to the right electrode, electrons moving right occupy higher electronic states than electrons moving left. Above a threshold bias voltage, a vibrational mode can be excited by the conduction electrons. This excitation causes the energy loss of the electrons. Since the electronic states moving right, at a lower energy, are occupied, electrons should move in the left direction; that means, electrons are scattered backwards. This backscattering causes the suppression in the differential conductance. This conductance suppression is viewed as a dip and a peak in *d*^2^*I*/*dV*^2^ curves at the positive and negative bias voltage, respectively.

**Figure 4:**
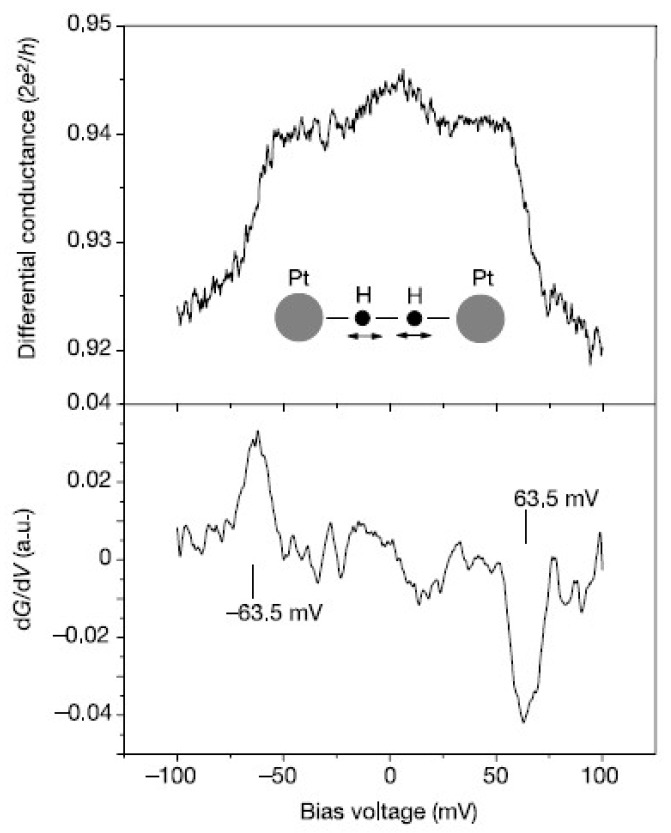
Differential conductance curve (**upper panel**) and its derivative (**lower panel**) for the hydrogen single-molecule junction with Pt electrodes measured at a zero bias conductance close to 1 *G*_0_. Reproduced with permission from [8], copyright Nature Publishing Group 2002.

Figure 4 shows the first PCS spectrum for the hydrogen single-molecule junction with Pt electrodes [8]. A symmetric downward step is observed around 60 mV in the differential conductance, and the corresponding dips are observed in its derivative (*d*^2^*I*/*dV*^2^). The shot noise, conductance fluctuation, and theoretical analysis reveal that a single hydrogen molecule bridges Pt electrodes with its molecular axis parallel to the junction axis. The observed dips in *d*^2^*I*/*dV*^2^ are assigned to the Pt–hydrogen vibrational mode by the theoretical calculation.

**Figure 5:**
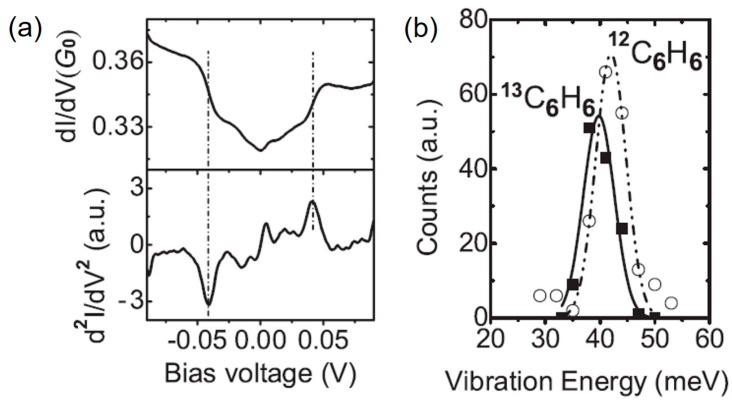
(**a**) Differential conductance curve (upper panel) and its derivative (lower panel) of the benzene single-molecule junction with Pt electrodes, measured at a zero bias conductance of 0*.*3 *G*_0_; (**b**) Distribution of vibrational energy for ^13^C_6_H_6_ and ^12^C_6_H_6_/Pt junctions. Reproduced with permission from [32] copyright Physical Review Letter Publishing Group 2008.

Figure 5 shows an IETS spectrum for a benzene single-molecular junction with Pt electrodes [32]. A symmetric upward step is observed around 40 mV in the differential conductance, and peaks are observed in *d*^2^*I*/*dV*^2^. The benzene single-molecule junction shows the isotope effect. Figure 5 shows the histogram of the peak energy in IETS for the single ^12^C_6_H_6_ and ^13^C_6_H_6_/Pt junctions. In the histogram, a peak is observed at 42 meV for the single ^12^C_6_H_6_/Pt junctions, and at 40 meV for the single ^13^C_6_H_6_/Pt junctions. Under the harmonic oscillator model, frequency (*ν*) of the vibrational mode is represented by

(1) $$\nu =k/\sqrt{m}$$

where, *m* and *k* are the molecular mass of benzene, and the spring constant of the benzene–Pt bond. Here, the benzene molecule is assumed to be much lighter than the Pt electrode. The mass ratio of ^13^C_6_H_6_ and ^12^C_6_H_6_ is 84/78. The vibrational energy for the ^13^C_6_H_6_/Pt junction is predicted to be 40 meV using the vibrational mode of 42 meV for ^12^C_6_H_6_/Pt junction. The predicted value of vibrational energy agrees with the experimental value, which supports that the observed peak in *d*^2^*I*/*dV*^2^ originates from the vibrational mode, and that benzene molecule bridges the Pt electrodes. The vibrational mode is assigned to be hindered-rotation mode (benzene–Pt) using the density functional theory (DFT) calculation.

**Figure 6:**
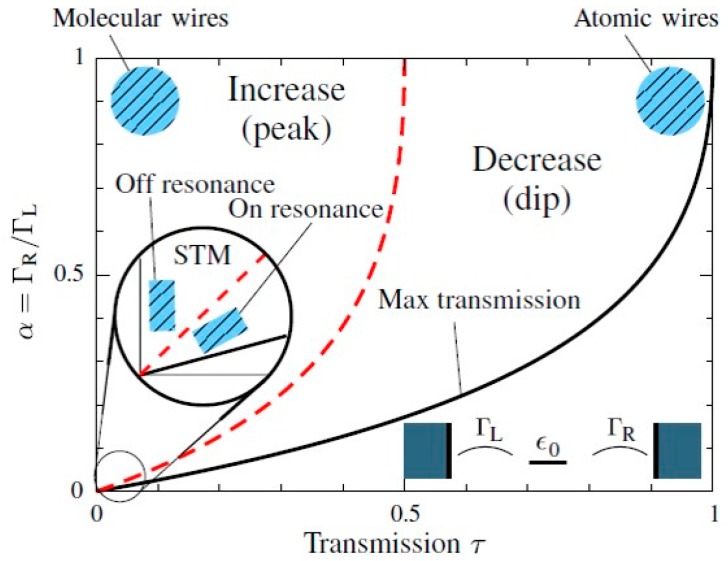
The sign of the conductance change as function of asymmetric factor (*α*) of metal–molecule coupling and transmission of the single-molecule junction, where electrons transport through a single channel. At a given *α*, the transmission (*τ*) has an upper limit *τ*_max_ (black curve), and the inelastic conductance change undergoes a sign change at *τ*_crossover_ = *τ*_max_/2 (red dashed curve). Reproduced with permission from [31] copyright Physical Review Letter Publishing Group 2008.

As discussed, the direction of change in the differential conductance by excitation of vibrational mode relies on the junction conductance. This topic has been theoretically analyzed by Paulsson et al. [31]. The calculated direction of the change in the differential conductance is shown in Figure 6 as a function transmission, and the asymmetry of the metal–molecule coupling. Here, electrons are assumed to be transported through a single channel. In the case of symmetric coupling (*α* = 1), the differential conductance increases by the vibrational mode excitation, if the conductance of the junction is below 0.5 *G*_0_ (tunneling regime). Meanwhile, when the conductance of the junction is above 0.5 *G*_0_ (contact regime), the conductance decreases by vibrational mode excitation. The experimental results for H_2_, H_2_O, ethylene, and benzenedithiol (BDT) junctions agree with this theoretical prediction [33,34,35,36].

**Figure 7:**
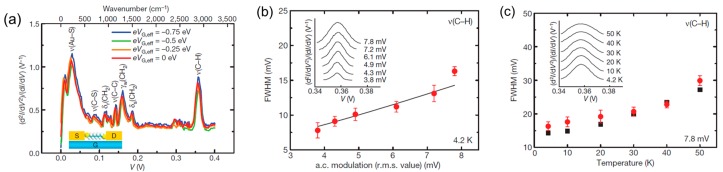
(**a**) IETS spectrum for the octanedithiol single-molecule junction; (**b**,**c**) Full width at half-maximum (FWHM) of the C–H stretching mode peak as a function of alternating-current (AC) modulation voltage and temperature. Experimental data are indicated by circles, and theoretical values are indicated by the solid line (**b**) and squares (**c**). Insets: examples of IETS spectra for different AC modulation voltage (**b**) and temperature (**c**). Reproduced with permission from [14,38] copyright Nano Letter Publishing Group 2008 and Nature Material Publishing Group 2006.

In the above examples, the molecule–metal vibrational modes are observed in the differential conductance spectra. The intramolecular vibrational mode can be also detected in the differential conductance spectra [37,38]. Figure 7 shows the IETS of the octane dithiol single-molecule junction. Vibrational modes are observed at 218, 694, 911, 1129, 1282, 1483, and 2879 cm^−1^, which are assigned to Au–S stretching, C–S stretching, C–H rocking, C–C stretching, C–H wagging, C–H scissoring, and C–C stretching [14]. The vibrational mode is the fingerprint of molecule, and the existence of vibrational modes verifies that the single octanedithiol molecule bridges Au electrodes. Song et al. showed the temperature and modulation voltage dependence of the full width at half-maximum (FWHM) of the IETS peaks, in order to confirm that the observed peak originated from the molecular vibrational mode [14]. The FWHM increases with the temperature and AC modulation voltage used for the lock-in detection. The FWHM of the peaks in the IETS is given by

(2) $$W={\left[{\left((1.7V_{m}\right)}^{2}+{\left(5.4k_{B}T/e\right)}^{2}+W_{I}{}^{2}\right]}^{1/2}$$

where *V_m_*, *k_B_*, *T*, and *W_I_* are the modulation voltage used for the lock-in detection, the Boltzmann constant, the temperature, and the intrinsic width [38]. The increase in FWHM with the temperature originates from the broadening of the Fermi distribution, and the increase in FWHM with the voltage modulation amplitude originates from the dynamic detection technique. The peaks in the differential conductance spectra are invisible above 100 K, due to the thermal broadening for the most single-molecule junctions. Therefore, differential conductance spectroscopy (PCS, IETS) requires low temperatures. The surface enhanced Raman scattering spectroscopy can be used even at room temperature, to get the vibrational mode of the single-molecule junction [39,40].

## 4. Electronic Structure 

**Figure 8:**
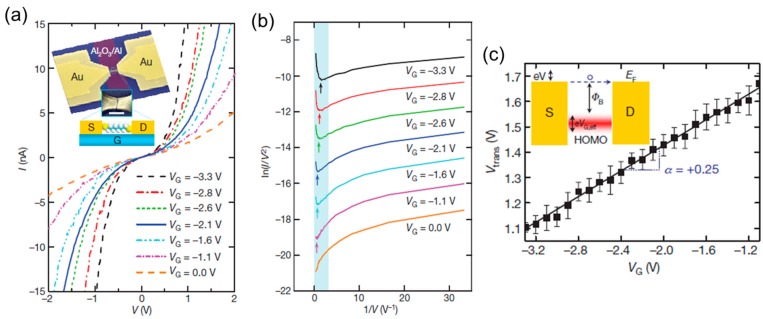
(**a**) *I*–*V* curves of the octanedithiol single-molecule junction measured with different gate voltages (*V*_G_). Inset, SEM image of single molecular transistor, *S*, *D*, *G* represent source, drain, and gate; (**b**) Fowler–Nordheim plots of the *I*–*V* curves for octanedithiol single-molecule junction; (**c**) The *V*_G_ -transition voltage (*V*_tran_) plot. Reproduced with permission from [14] copyright Nature Publishing Group 2009.

The electronic structure around the Fermi level can be determined by the *I*–*V* measurement [41]. The *I*–*V* curves can be analyzed based on two different models; single channel transport model [42] and effective tunneling barrier model (i.e., Simmons model) [43,44]. The effective tunneling barrier model has been successfully applied to tunneling though highly insulating oxide films [45] and adapted to tunneling though molecular junctions [44,46,47,48]. The *I*–*V* fit and analysis based on the Simmons model did not always work well for the molecular junctions [44,47,48]. In contrast to the insulating oxide films, there exists relatively strong electronic coupling between molecules and metals in molecular junctions. As a result, fitting molecular junction-data to the Simmons model becomes ambiguous, because the fit parameters such as barrier height, barrier width, and contact area can have physically unreal values [47]. Beside the *I*–*V* fitting approach based on the Simmons model, transition voltage spectroscopy (TVS) tuned out to be useful for determining effective tunneling barrier height of molecular junctions [41,49,50]. In the TVS, *I*–*V* curves is replotted as ln(*I*/*V*^2^) vs. 1/*V*, the so-called Fowler–Nordheim plot [49]. The TVS is based on transition of the charge transport mechanism from tunneling to field emission as the bias voltage increases. The minimum in the *F*–*N* plot transition corresponds to the transition, and the minimum position is named the transition voltage, *V*_tran_. The previously combined ultraviolet photoemission spectroscopy (UPS) and TVS study revealed that *V*_tran_ is proportional to the energy difference between the Fermi level of metal electrode and conduction orbital [50]. Song et al. applied TVS to the single-molecule transistor [14]. Figure 8 shows TVS for the octanedithiol single-molecule junction with different gate voltages. The *V*_tran_ increases with the gate voltage. At the negative gate voltage, highest occupied molecular orbital (HOMO) approaches the Fermi level, which causes the high conductivity of the junction. The working mechanism of the p-type single molecular transistor is revealed by TVS.

In the single channel model, the transmission curve (transmission probability as a function of energy) of single-molecule junction is represented by the Lorentzian-type function (Equation (3))

(3) $$\tau (E)=\frac{4\Gamma_{L}\Gamma_{R}}{{\left(\Gamma_{L}+\Gamma_{R}\right)}^{2}+{\left(E-\varepsilon \right)}^{2}}$$

where *Γ* is the electronic coupling between molecule and electrodes, and *ε* is the molecular orbital energy relative to the Fermi level. Here, *α* = *Γ*_L_/*Γ* (*Γ* = *Γ*_L_ + *Γ*_R_) is the symmetric parameter. Using Equation (3), the electric current is calculated, and it is represented as the arctangent-type function (Equation (4))

(4) $$I(V)=\frac{8e}{h}\alpha (1-\alpha )\Gamma \left\{\tan^{-1}\left[\frac{\alpha eV-\varepsilon }{\Gamma }\right]+\tan^{-1}\left[\frac{(1-\alpha )eV+\varepsilon }{\Gamma }\right]\right\}$$

**Figure 9:**
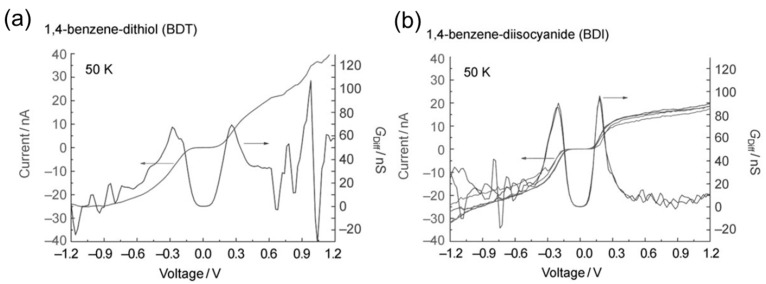
The *I*–*V* curves and corresponding transmission curves of (**a**) 1,4-benzene–dithiol; and (**b**) 1,4-benzene–diisocyanide single-molecule junctions. Reproduced with permission from [51] copyright ChemPhysChem Publishing Group 20011.

**Figure 10:**
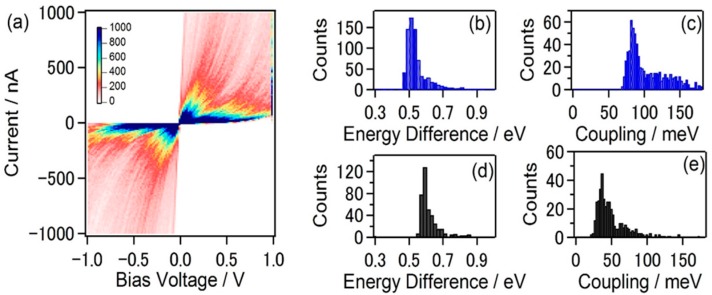
(**a**) 2D histogram of the *I*–*V* curves of C_60_ single molecule junction; The distribution of (**b**,**d**) *ε* and (**c**,**e**) *Γ*. The fitting results of *ε* and *Γ* are separately shown for the high conductance state (**b**,**c**) and low conductance state (**d**,**e**) in the figures. Reproduced with permission from [52] copyright Chemistry—An Asian Journal Publishing Group 2017.

Figure 9 shows the example of *I*–*V* curves of the di-substituted benzene single-molecule junctions with different anchoring groups [51]. By fitting arctangent-type function (Equation (4)) with the measured *I–V* curves, *Γ* and *ε* were determined. The *ε* was 266 meV for BDT and 197 meV for BDI (benzene–diisocyanide). The difference in *ε* is explained by the electron affinity of the molecule. The *Γ* was 135 meV for BDT, and 95 meV for BDI. The larger *Γ* for BDT is explained by the formation of the strong covalent S–Au bond in the BDT single-molecule junction. The difference in the electronic structures between BDT and BDI single-molecule junction was evaluated by *I*–*V* curves. Komoto et al. reported the electronic structures of two distinct conductance states for C_60_ single-molecule junctions by *I*–*V* measurements [52]. Figure 10a shows the distribution of *I*–*V* curves for the C_60_ single-molecule junctions. Two distinct states are visible in the 2D *I*–*V* histogram. The *ε* and *Γ* were determined to be *ε* = 0.51 eV and *Γ* = 0.082 eV for the high conductance state, and *ε*_0_ = 0.59 eV and *Γ* = 0.038 eV for the low conductance state. These results indicate that the conductance difference is mainly caused by the difference in *Γ*. 

## 5. Interface Structure

**Figure 11:**
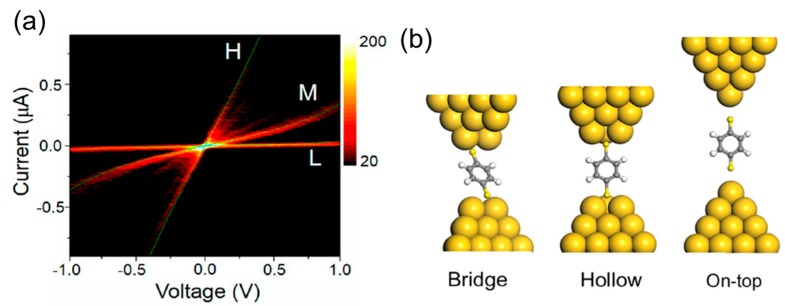
(**a**) Distribution of *I*–*V* curves of benzenedithiol (BDT) junctions constructed from 200 *I*–*V* curves; (**b**) Structural model of BDT single-molecule junctions. Bridge, hollow, on-top are assigned to *H*, *M*, and *L* states in *I*–*V* curves. Reproduced with permission from [40,53] copyright Science Report Publishing Group 2016 and Journal of the American Chemical Society Publishing Group 2016.

The metal–molecule coupling strength obtained by the *I*–*V* analysis is sensitive to the metal–molecule interface structure of the single-molecule junction. The interface structure of the single-molecule junction can be thus determined, based on the analysis of the strength of the metal–molecule coupling. Figure 11a is the distribution of *I*–*V* curves of the BDT single-molecule junctions [40,53]. Three conductance states are clearly distinguishable. Each conductance state is labelled as high (H), medium (M), and low (L) conductance state, according to conductance. By fitting the *I*–*V* curves with Equation (4), the *Γ* and *ε* values are determined to be 0.12 eV and 0.83 eV for *H*, 0.05 eV and 0.87 eV for *M*, and 0.013 eV and 0.76 eV for *L* state, respectively. The average conductance (*G*) is 0.02 *G*_0_, 3 × 10^−3^ *G*_0_, and 3 × 10^−4^ *G*_0_ for *H*, *M*, and *L* state, respectively. Three physical parameters of the single-molecule junction, *Γ*, *ε*, and *G*, can be obtained by analyzing the *I*–*V* curves. The theoretical calculation with the model clusters having different interface structures also provides *Γ*, *ε*, and *G*. By comparing these parameters between the experimental and theoretical results, the *L*, *M*, *H* conductance states in *I–V* curves are assigned to interface structures of the on-top, hollow, bridge site adsorption, respectively [53].

## 6. Type of Charge Carriers

Thermoelectric voltage is induced in response to a temperature gradient across the materials by the Seebeck effect. Recently, thermoelectric power generators, which directly convert heat flux to electric energy, have attracted attention, because they can convert waste heat into useful electric power. The previously reported theoretical study predicts large thermopower for the low dimensional material. Thermopower of the single-molecule junction is currently one of the hot topics [54,55]. Thermopower also provides useful information about the single-molecule junction. The signs of thermopower, plus or minus, are associated with the type of major carriers, electrons, or holes. In other words, the thermopower measurements decide whether the electrons flow through the single-molecule junction via LUMO, or the hole flow via HOMO. As mentioned above, *I*–*V* characteristics can determine the energy difference between the conduction orbital and Fermi level. Thus, combining *I*–*V* and thermopower measurements, we can determine the electric structure of the single-molecule junction near the Fermi level.

**Figure 12:**
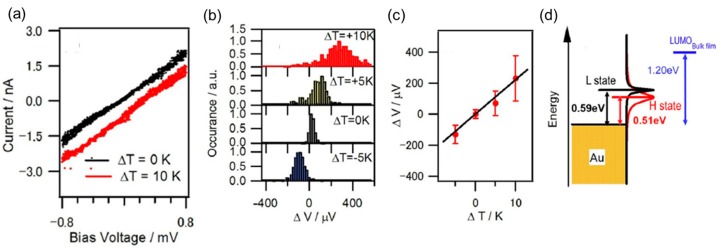
(**a**) Example of the *I*–*V* characteristics of the C_60_ single-molecule junction at a temperature difference of 0 and 10 K; (**b**) Histograms of the thermoelectric voltage (Δ*V*) at temperature differences (Δ*T*) of −5 K, 0, +5 K, and +10 K; (**c**) Plot of the peak values in the of the Δ*V* histogram as a function of Δ*T*; (**d**) Energy level diagram of the high and low conductance states of the C_60_ single-molecule junction. Reproduced with permission from [52] copyright Chemistry—An Asian Journal Publishing Group 2017.

The thermopower of the single-molecule junction can be observed as the offset voltage of *I*–*V* characteristics, as seen in Figure 12a. Figure 12a shows the *I*–*V* curve of the C_60_ single molecule junction measured at different temperature difference [52]. The offset voltage of ~0.1 mV was observed at a temperature difference of 10 K, while offset voltage was 0 at a temperature difference of 0. Figure 12c shows the averaged offset voltage as a function of the temperature difference. From the slope in Figure 12c, thermoelectric power of the C_60_ single molecule junction, was determined to be −12.6 μV/K. The positive slope corresponds to a negative thermoelectric power, which means that LUMO was closer to the Fermi level than HOMO. The type of charge carriers (conduction orbital) can be determined by the *I*–*V* measurement. The determination of type of charge carrier has been reported for various systems (e.g., bipyridine, oligophenyls, alkanes) using *I*–*V* measurements [56,57]. 

In the case of C_60_ single-molecule junction, the energy difference between conduction orbital and Fermi level was determined to be 0.5~0.6 eV by the *I*–*V* measurement, as discussed above. The thermopower measurement revealed that the conduction orbital was LUMO. By combining the results of the *I*–*V* and thermoelectric measurements, the electronic structure of the C_60_ single-molecule junction was determined as shown in Figure 12d.

## 7. Spin State

**Figure 13:**
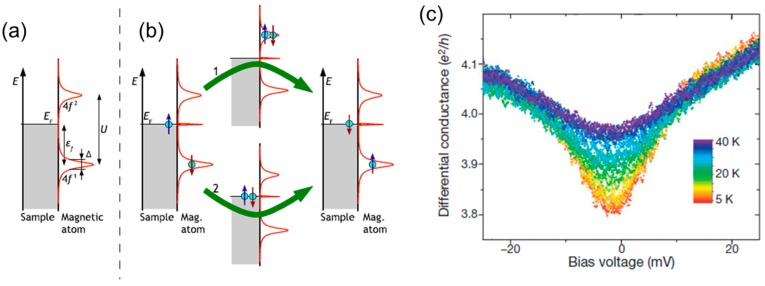
(**a**) Energy-dependent density of state (DOS) for the system where a single magnetic atom adsorbs on a metal sample; (**b**) The spin-flip process induced by the interaction between of the singly occupied state and bulk electron of opposite spin. The spin-flip process (process 1 and 2) form the Kondo resonance around the Fermi energy (**c**) Differential conductance curves for the Co atomic junction measured at different temperatures. Reproduced with permission from [59] copyright Nature Publishing Group 2017.

When localized spin is present in the single-molecule junction, a sharp peak or dip, the so-called Kondo peak, can appear around the zero bias regime in the differential conductance curves [26]. The spin state of the single-molecule junction can be, thus, evaluated by analyzing the Kondo peak in the *I*–*V* curve. The Kondo effect is usually investigated by the temperature dependence of the electronic conductance. In conventional metal, electronic conductance increases with a decrease in temperature. When magnetic impurities are present in a metal, the conductance decreases with a decrease in temperature below a certain temperature. The abnormal conductance decrease originates from the interaction of the spin of the conduction electrons with the localized spin of the magnetic impurities. The spin excitation state is created around the Fermi energy due to the Kondo effect, as shown in Figure 13. Figure 13b shows the creation of the spin excitation state for a single magnetic atom (4*f* metal) on a metal surface [26]. A singly occupied 4*f* state (4*f*^1^) is formed below the Fermi energy. The 4*f*^1^ state is separated from the 4*f*^2^ state, where two electrons with opposite spins are occupied, by the Coulomb repulsion energy (*U).* The spin of the singly occupied 4*f*^1^ state could be flipped by conduction electrons with opposite spin through process 1 or 2. In the case of the process 1, electron is first removed from the 4*f*^1^ state, and then is refiled. In Process 2, the second electron is first put into the 4*f*^1^ state, and then one electron is removed from the 4*f*^2^ state. The spin state of the final 4*f*_1_ state is opposite comparing with the initial 4*f*^1^ state. This spin-flip process creates the Kondo resonance around the Fermi level, which causes the Fano-Kondo resonance peak or dip in the differential conductance curves. The Fano-Kondo resonance is represented by

(5) $$\frac{dI}{dV}=g_{0}+\frac{A}{1+q^{2}}\frac{{\left(q+\epsilon \right)}^{2}}{1+\epsilon^{2}},\epsilon =\frac{eV-\epsilon_{s}}{k_{b}T_{K}}$$

where $g_{0}$ is flat component, $T_{K}$ is Kondo temperature, *q* is dimensionless Fano parameter, *A* is amplitude of the feature, and $k_{b}$ is Boltzmann’s constant [58]. The Kondo temperature is a characteristic temperature and determines the peak width of the Kondo resonance in energy dependent density of state (DOS). The Kondo temperature provides the strength of the interaction between magnetic moment of magnetic impurity and conduction electrons. 

Figure 13 shows the differential conductance curves of the Co atomic junction as a function of temperature [59]. Kondo temperature is determined to be 120 K by fitting the differential conductance curve with Equation (6). The amplitude of the Fano feature decreases with the temperature. The decrease in amplitude is caused by the smearing of the Fermi–Dirac distributions of the initial state and final states during the spin-flip transition via the intermediate state. It is noteworthy that the atomic junction of a pure Co (ferromagnet) unexpectedly reveals the Kondo effect, although Co is ferromagnetic material in bulk. The theoretical calculation shows that *d* electrons antiferromagnetically couple with the sp conduction electrons in the ferromagnet nano contact. Therefore, the localized magnetic moment is screened by the conduction electrons, which causes the Kondo effect in the Co atomic junction. As for the bulk, sp−d hybridization vanishes, owing to the small anisotropy of the crystal environment. The Kondo effect and Zeeman splitting have been investigated for various single-molecule junction and metal atomic junction, in order to discuss the spin state of the single-molecule junction [60,61].

Kondo effect can be seen in single-molecule junction with a transition metal complex of Co(tpy-SH)_2_ (tpy-SH is 4′-mercapto-2,2′:6′,2″-terpyridine) [60,61]. The Co complexes have spin *S* = 1. Without stretching the junction, *dI*/*dV* spectrum exhibit a single peak centered at *V* = 0, which is the signature of Kondo-assisted tunneling through the molecule. As the junction is stretched, the Kondo peak splits into two peaks for the change in electrode spacing that varied from device to device. The splitting peak is caused by a higher-spin *S* = 1 Kondo effect, together with the breaking of degeneracy among the *S* = 1 triplet ground state by molecular distortion. For an unstretched *S* = 1 ion in a ligand field with octahedral symmetry, the triplet states are strictly degenerate. When the molecule is stretched, the *Sz* = 0 state is lowered by a zero-field splitting energy below the *Sz* = ±1 states. This broken degeneracy quenches the Kondo resonance near *V* = 0 and causes conductance peaks because of inelastic tunneling.

**Figure 14:**
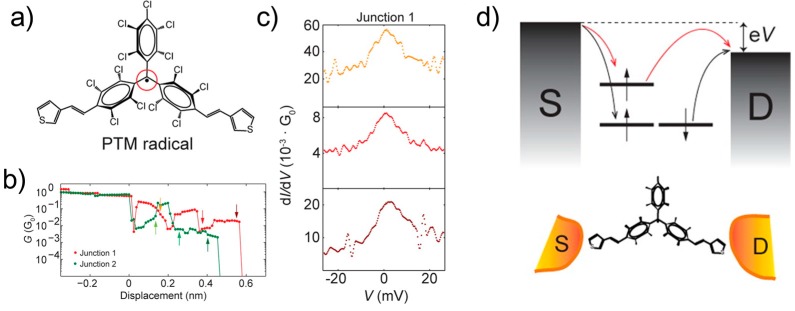
(**a**) Polychlorotriphenylmethyl (PTM) radical molecule; (**b**) Conductance traces for two different PTM molecular junctions; (**c**) *dI*/*dV* spectra measured at displacements indicated by the arrows in (**b**); (**d**) Schematic image of the energy levels/orbitals involved in the electron transport through the PTM molecule. Reproduced with permission from [60], copyright Nano Letter Publishing Group 2015.

The Kondo effect is also observed for the organic radical single-molecule junction. Riccardo et al. reported the Kondo effect in a polychlorotriphenylmethyl (PTM) radical single-molecule junction. The PTM is all-organic composition without transition metal [60]. Figure 14 shows the conductance traces of the two different samples together with the *dI*/*dV* spectra. The *dI*/*dV* spectrum shows a zero-bias anomaly corresponding to the Kondo effect. Figure 14d is the schematic image of electron transport through the PTM single-molecule junction. Electrons transport via a spin unpolarized transport channel (HOMO) and spin-flipping Kondo-mediated transport channel. The Kondo mediated channel arises from the singly occupied molecular orbital (SOMO). Here, it is notable that the Kondo peak is not sensitive to the displacement (Figure 14c). The SOMO is localized on the radical carbon atom protected by three chlorinated phenyl rings, indicating a weak hybridization of the atomic orbital and its strong atomic character. The strong localization of the SOMO makes the Kondo state stable.

## 8. Conclusions

In this mini review, we describe examples of *I*–*V* characteristics of single-molecule junction. The physical parameters obtained by the *I*–*V* curve give us information about the diode characteristics, vibrational movements, type of charge carriers, atomic and electronic structures, and spin state of the single-molecule junction. Currently, various single-molecule devices are developed, such as switch, memory, transistor, sensor, diode, light source, and thermopower generators. For practical use, the single-molecule junction should be well defined. The information obtained by *I*–*V* curves is essential for the characterization of the single-molecule junction. The working principle of the single molecular devices will become clear using this information, which enables the development of more advanced single molecular devices. New physical and chemical properties can be also discovered based on the information obtained by the *I*–*V* curves of single-molecule junctions.
